# Antiepileptic Effect and Safety Profile of Rapamycin in Pediatric Patients With Tuberous Sclerosis Complex

**DOI:** 10.3389/fneur.2022.704978

**Published:** 2022-04-29

**Authors:** Krzysztof Sadowski, Kamil Sijko, Dorota Domańska-Pakieła, Julita Borkowska, Dariusz Chmielewski, Agata Ulatowska, Sergiusz Józwiak, Katarzyna Kotulska

**Affiliations:** ^1^Department of Neurology and Epileptology, Children's Memorial Health Institute, Warsaw, Poland; ^2^Transition Technologies, Warsaw, Poland; ^3^Department of Child Neurology, Medical University of Warsaw, Warsaw, Poland

**Keywords:** tuberous sclerosis, epilepsy, rapamycin, sirolimus, mTOR inhibitors

## Abstract

**Background:**

Epilepsy develops in 70–90% of children with Tuberous Sclerosis Complex (TSC) and is often resistant to medication. Treatment with mTOR pathway inhibitors is an important therapeutic option in drug-resistant epilepsy associated with TSC. Our study evaluated the antiepileptic effect of rapamycin in the pediatric population of patients diagnosed with TSC.

**Methods:**

This single center, open-label study evaluated safety and anti-epileptic efficacy of 12 months of rapamycin treatment in 32 patients aged from 11 months to 14 years with drug-resistant TSC- associated epilepsy.

**Results:**

After the first 6 months of treatment, the improvement in seizure frequency, defined as at least a 50% reduction in the number of seizures per week compared to baseline, was seen in 18 individuals (56.25%). We observed no change in 12 individuals (37.5%) and worsening, defined as increase in the number of seizures—in 2 patients (6.25%). The overall improvement defined as at least a 50% reduction in seizure frequency was found in 65.6% of all patients after 12 months with 28% of patients obtaining complete remission. Another five patients experienced at least an 80% reduction in the frequency of seizures. Concomitant treatment with vigabatrin, and to a much lesser extent topiramate and levetiracetam, was an additional favorable prognostic factor for the success of the therapy. A linear relationship between the cumulative dose of rapamycin and its therapeutic effect was observed. The safety profile of the drug was satisfactory. In none of the observed cases did the adverse events reach the level that required withdrawal of the rapamycin treatment. The reason for dropouts was insufficient drug efficacy in 3 cases.

**Conclusions:**

Long-term use of rapamycin, especially in combination with vigabatrin, might be a beneficial therapeutic option in the treatment of drug-resistant epilepsy in children with TSC.

## Introduction

Tuberous sclerosis complex (TSC) is an autosomal dominant, multisystem genetic disorder affecting 1 in 6,000 individuals (1). TSC is caused by the inactivation of either of the tumor suppressor genes *TSC1* (locus 9q34) or *TSC2* (locus 16p13.3) (2, 3). Their protein products, hamartin and tuberin, respectively, form a heterodimer (TSC1–TSC2 complex) and play an important role in the regulation of cell proliferation and differentiation processes through negative mTOR (mechanistic target of rapamycin) pathway regulation (3, 4). The tuberin-hamartin heterodimer acts as a complex inhibiting mTOR signaling pathway. mTOR disinhibition leads to tumor or hamartoma formation in multiple organs. The hallmark of the disease is the formation of benign tumors in the brain, kidneys, liver, heart, retina, lungs, and skin. Central nervous system lesions include cortical dysplasia, subependymal nodules (SENs), and subependymal giant cell astrocytomas (SEGAs) (5). Epilepsy is the most frequent neurological symptom of TSC, affecting about 80–90% of patients (6). Focal seizures and epileptic spasms are the most prevalent seizure types (7, 8) and about two-thirds of them are refractory to medication. In the vast majority (80–90%) of cases, the first seizures appear before 2 years of age, with 63 to 73% of patients having seizures during the first year of life (9). Drug-resistant epilepsy, especially in infants, is the strongest risk factor for intellectual disability in later life (10, 11). Vigabatrin (VGB) is a gold standard first-line treatment for TSC, associated infantile spasms, and/or focal seizures (12, 13). When VGB monotherapy fails, a combination of other antiepileptic drugs, primarily GABAergic AEDs, is recommended. The management of TSC-associated refractory seizures, aside from standard AEDs, includes a ketogenic diet, epilepsy surgery, and vagus nerve stimulation (VNS). However, existing treatments are often ineffective and >60% of patients develop treatment-refractory seizures, in contrast to about 30% of patients with epilepsy without TSC (14–17). Given that dysregulation and hyperactivation of the mTOR pathway are for the most part responsible for the pathophysiology of TSC, mTOR inhibitors were introduced for the treatment of various manifestations of TSC. Everolimus, a rapamycin derivate, has been approved by regulatory authorities in Europe and the US for treatment of TSC-associated SEGAs, kidney angiomyolipomas (AMLs), and epilepsy (18–20). Less evidence is available about the therapeutic effects of rapamycin (21–23). Preliminary studies revealed a satisfactory safety profile for rapamycin in TSC individuals (22–24). However, the data on its efficacy are conflicting, mainly due to the small groups of patients treated and short-term follow up (25, 26). There are no studies directly comparing everolimus and rapamycin in TSC. The availability of both drugs differs in individual countries. For example, in Poland, everolimus is not reimbursed by the national healthcare service for TSC-associated epilepsy, contrary to rapamycin, which is reimbursed for TSC, regardless of its manifestations. Despite its reimbursement by the national healthcare service in Poland, the treatment with rapamycin is regarded as experimental. In this paper, we present the results of an open-label single-center study of the safety and efficacy of rapamycin in pediatric patients with TSC-related epilepsy.

## Patients and Methods

### Patients

This is an open-label, single-center study to evaluate the efficacy and safety of rapamycin in TSC- associated epilepsy in children. Children aged between 6 months and 18 years with a definite diagnosis of TSC according to the consensus criteria (5), were preliminarily considered to be eligible for inclusion in the study. Inclusion criteria were: the diagnosis of drug-resistant epilepsy, defined as failure of seizure control with at least two adequate AEDs in therapeutic doses (27) and at least one seizure per week within the last 8 weeks preceding rapamycin introduction (as assessed on the basis of seizure diaries provided by the caregivers). Exclusion criteria were comprised of: infantile spasms at baseline, severe renal dysfunction, recent surgery (up to 8 weeks before inclusion), active infection, documented hypersensitivity to mTOR inhibitors in the past, and any diseases or other circumstances deemed to impede participation in therapy. Patients were also excluded if they had had prior systemic treatment with an mTOR inhibitor. Caregivers' written informed consent was required in each case before the introduction of therapy. All therapeutic procedures were performed in agreement with the Declaration of Helsinki (2008) and Good Clinical Practice guidelines as well as all national and local regulations. The study was approved by the local Ethics Committee at The Children's Memorial Health Institute, Warsaw, Poland.

### Study Design

All participants were followed prospectively and intended to receive rapamycin treatment for 12 months. Blood samples were collected: during the introduction of the therapeutic dose, 1 week, 2 weeks, and 1 month after starting rapamycin, at checkpoints and when required by the patient's clinical state. Checkpoints for assessing treatment efficacy were scheduled after 6 and 12 months of treatment. Any adverse events that occurred during the treatment have been documented and evaluated according to the WHO adverse reaction terminology and the National Cancer Institute common terminology criteria for adverse events version 3.0. Patients received a widely available pharmaceutical formulation of rapamycin (Rapamune; Pfizer). Depending on the patient's age and individual conditions and needs, the formulation of the drug was administered in the form of an oral solution (1 mg/ml) or tablets (1 mg). Children received a starting dose of rapamycin based on their body surface area, ranging from 0.5 mg/m^2^ for infants and children aged <3 years, 1 mg/m^2^ for children aged from 3 to 10 years, 2 mg/m^2^ for teenagers. Rapamycin dosage was titrated to blood trough levels between 5 and 10 ng/mL. In case of adverse events of grade 2 or higher, the rapamycin treatment was discontinued until the adverse event subsided or reached grade 1 and was restarted at the last dose without adverse events. In case of grade 3 adverse events permanent drug discontinuation was undertaken.

### Outcome

The outcome endpoints were analyzed at 6 and 12 months of treatment. The assessment of the effectiveness of the antiepileptic treatment with rapamycin was based on self-reporting provided by parents in the form of diaries. The seizure number per week at 6 and 12 months was compared with baseline values, which were determined based on the analysis of diaries covering the last 8 weeks before the rapamycin treatment. Improvement was defined as at least 50% reduction in the frequency of seizures compared to baseline.

### Statistical Analysis

The analysis was performed using R version 3.6.2 (https://www.R-project.org/). The packages used for visualization and general-analysis were: tidyverse; ggmosaic (https://CRAN.R-project.org/); readxl (https://CRAN.R-project.org/package=readxl); stringi (28); lme4 for Linear Mixed-Effects Models (LME) models (29); h2o for machine-learning exploration and interpretation (https://CRAN.R-project.org/package=h2o); and DALEX (30). Entries were coerced to a numerical range (min-max) per weekly basis where a precise estimate was not available or where there were significant differences between weeks (for example, in the case of infection). In the majority of analyses, the middle point of the range was taken as the best estimate, and the plots present the whole range as well. Mathematical modeling with the application of Linear Mixed-Effects Models was used to assess the impact of preceding antiepileptic therapies on the potential efficacy of rapamycin. The predicted variable was the percentage improvement in seizure frequency with the first measurement (timepoint “A”) taken as the reference. For example, patient 22 who started with an average of 3 seizures per week, presented with 1.5 seizures per week after 6 months on rapamycin and to 2 after 12 months. There are three values for this patient in the dataset—the first value of improvement is 0 (at baseline), then 50 after 6 months, and finally 33.3 after 12 months. Explanatory variables were assigned to two groups: fixed variables for all three measurements and change variables for between the timepoints. The fixed variables included: gender; concomitant antiepileptic drugs as binary indicators (LEV, LTG, TPM, VGB, VPA, Other); mutation (TSC1, TSC2, no mutation identified, no data); indicator of the onset of seizures (first seizures up to 90th day of life or after); time with seizures before enrolment in the study. The change variables included: the time-point and the cumulative rapamycin dose. The improvement was modeled first with the Linear Mixed-Effects Models: the model was informed about the design of the study: provided patient id grouped data appropriately.

Overall, four models were computed:

0. the empty (baseline) model, containing the specification of the study design only (the measurements nested within patients)
1. the rapamycin model containing information about the cumulative rapamycin intake only
2. the model that contained additional information about the binary indicators of concomitant medication
3. the model containing all the remaining variables.

We compared these models using ANOVA to assess whether improvements in any particular model fitted the compensated additional degrees of freedom (DF) used for additional variables in the modeling. Additionally, we applied a machine-learning algorithm to the same dataset using the AutoML procedure from h2o package with the default parameters set. The best model was an ensemble of multiple ML algorithms with a mean average error (MAE) of 27%. Variable importance analysis and partial dependence plots (PDP) for important variables (30) were employed.

## Results

The main clinically relevant findings of this study are as follows:

- In a 12-month observation period, rapamycin proved to be effective (≥50% reduction of seizure frequency) for 65.5% of patients
- The safety profile proved to be favorable in all age groups
- A linear relationship between the cumulative dose of rapamycin and its therapeutic effect was observed

Below is a detailed description and interpretation of the results obtained in the study.

### Patients

Between January 2016 and January 2019, 60 TSC children received reimbursed treatment with rapamycin at The Children's Memorial Health Institute, Warsaw, Poland. Thirty-five of them, 17 females and 18 males, met the inclusion criteria for this study. The median age of patients at the time of rapamycin introduction was 4 years and 1 month and varied from 11 months to 14 years and 3 months. Thirty-two patients (18 males and 16 females) received rapamycin for at least 12 months. The efficacy and safety data were analyzed for patients who completed the study. The reason for dropouts was insufficient drug efficacy in all three cases. Basic demographic and clinical data are summarized in Table 1. The study group was almost homogeneous in terms of the type of seizures observed, with a distinct predominance of focal seizures with impaired awareness. Patients with infantile spasms were excluded from the study. Among the concomitant antiepileptic drugs, vigabatrin (84%), valproic acid (69%), and topiramate (31%) were the most commonly used in the study group (Table 1).

**Table 1:** Baseline demographics and medical history characteristics.

Age at inclusion [years, mean (range)]	5.2 (0.9–14.3)
Male/female [number, (percentage)]	16/16 (50/50)
Mutation TSC1/TSC2/NMI/no data [number (percentage)]	3/18/1/10 (9/56/3/32)
Age at first seizure [months, mean (range)]	6.6 (0–30)
Seizure frequency per day at baseline [mean (range)]	8 (1–10)
No. of AEDs at baseline [median (range)]	3 (1–4)
AEDs usage at baseline [no of patients]
VGB	28
VPA	23
TPM	10
LTG	9
LEV	8
CLB	5
OXC	1

*Data in brackets are range (“-“) and % (“/”). NMI, no mutation identified; AEDs, antiepileptic drugs; VGB, vigabatrin; VPA, valproic acid; TPM, topiramate; LTG, lamotrigine; LEV, levetiracetam; CLB, clobazam; OXC, oxcarbazepine*.

### Treatment

The mean daily dose of rapamycin in the first 6 months of treatment was 1.1 mg/day and ranged from 0.4 to 3 mg/day. In the next 6 months of treatment, the mean daily rapamycin dose was 1.3 mg/day and varied between 0.4 and 3 mg, respectively.

### Epilepsy and Treatment Outcome

**Figure 1:**
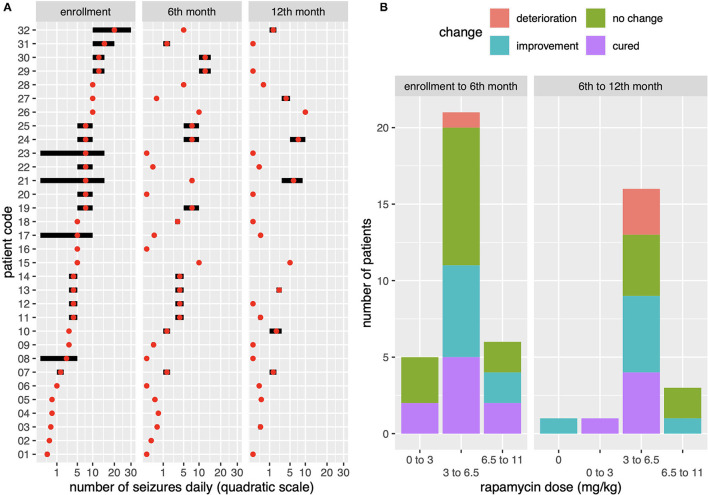
**(A)** Presentation of changes in seizure dynamics in individual patients at baseline (A) and in consecutive semi-annual control points (B, C) on a quadratic scale (1a). Code symbols for individual patients on the vertical axis. On the horizontal axis, the daily seizure frequency is scaled in the range 0–5–10–20–30 seizures per day. The score marks correspond to the seizure frequency for each patient and illustrate the dynamics of treatment efficacy at 6 months and 1 year after rapamycin initiation. **(B)** rapamycin concentration distributions for the entire population studied in relation to the therapeutic effect obtained in first 6 months (AB) then after another 6 months (BC) that means after 1 year of rapamycin therapy. The achieved therapeutic effect, defined as improvement in seizure control, is compared to the taken dose of the drug per kg with the observed optimum in the range of 3–6.6 mg/kg.

**Figure 2:**
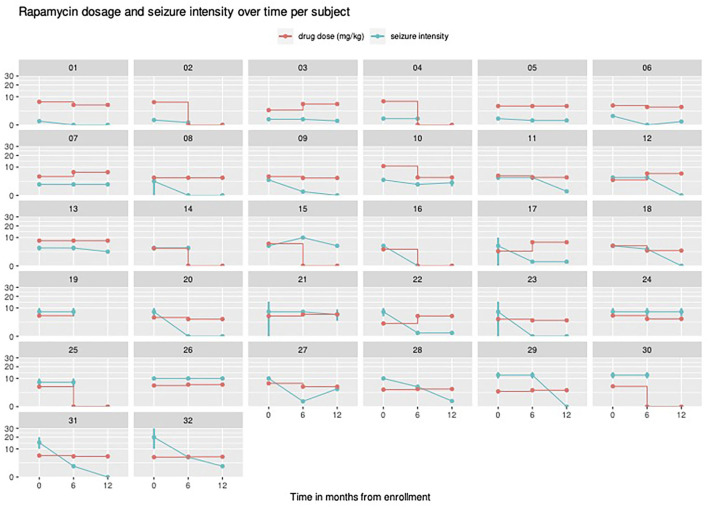
Individual therapy regimes (rapamycin dosage) visualized for all patients across the study together with observed seizure intensity. If seizure intensity was described by the caregivers as interval it was visualized as a vertical bar. The vertical axis shows the serum rapamycin concentration ranges, marked for individual patients with red lines. Seizure frequency was quantified by the course of the green lines—decrease with lower frequency and increase with higher frequency of seizures. “Seizure intensity” was defined as seizure number per week.

At baseline, focal seizures with impaired awareness were found in 24 individuals, tonic seizures in six individuals, atonic seizures in three, and focal to bilateral tonic-clonic seizures in three patients, respectively. Polymorphic seizures were observed in seven children. The mean frequency of seizures was 8 per day (Table 1). EEG at baseline disclosed focal discharges in 30 cases and generalized discharges in 15 cases. Thirteen patients presented with both focal and generalized abnormalities. Hypsarrhythmia was not observed in this group. At the end of follow-up, after 12 months of rapamycin treatment, five patients showed normalization of previously abnormal EEG recordings; in 26 patients, the EEG features remained unchanged; and one patient developed generalized discharges. After the first 6 months of treatment, the improvement in seizure frequency, defined as at least a 50% reduction in the number of seizures per week compared to baseline, was seen in 18 individuals (56.25%), no change was seen in 12 individuals (37.5%) and worsening, defined as increase in the number of seizures—was seen in two patients (6.25%). After the next 6 months, another three patients showed improvement. Together with the sustained improvement in the first group of responders, a clinical improvement of the level of 65.6% was noted. At 12 months, no patient reported a worsening in seizure frequency. Figure 1 depicts outcome patterns in observed patients. After 1 year of follow-up, complete remission of seizures was found in nine patients. Another five patients experienced at least an 80% reduction in the frequency of seizures. The impact of rapamycin blood concentrations and their dynamics over time on epilepsy in individual patients was assessed (Figure 2). Modeling the dependence of the therapeutic effect achieved at checkpoints on the drug concentration in blood serum revealed a curvilinear relationship, with an optimum observed at the level of 4.5 ng/ml/day. At the same time, the relationship between the therapeutic effect and the cumulative total dose of rapamycin appeared to be linear, which suggests greater benefits from long-term rapamycin intake. Across all model estimations for rapamycin, the cumulative dose had a value of around 0.033 per 1 mg consumed (e.g., a person who took 1,000 g of rapamycin would have expected 33% higher improvement).

**Figure 3:**
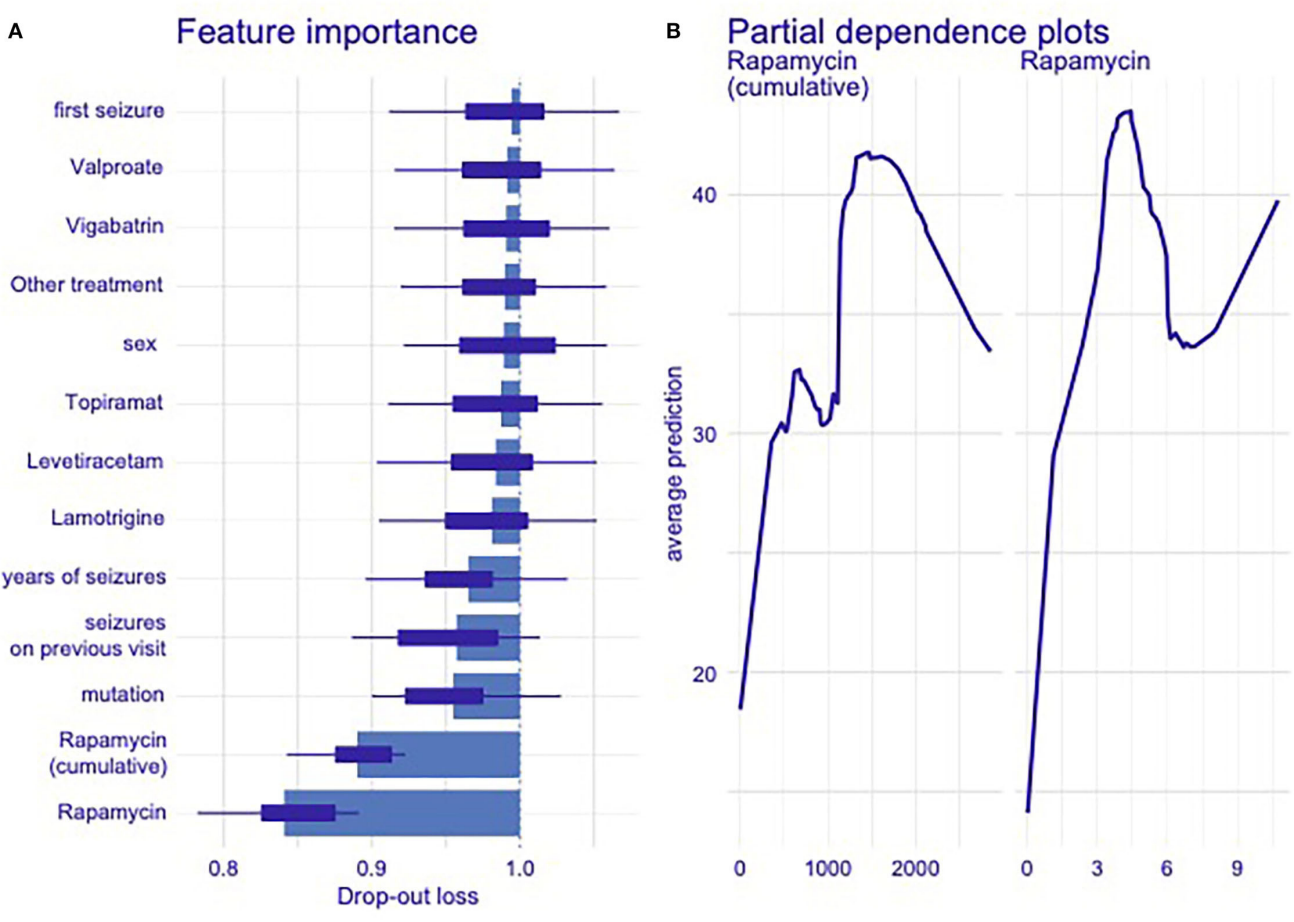
**(A,B)** Evaluation of the potential influence of additional demographic and pharmacological factors on the efficacy of rapamycin therapy **(A)** and evaluation of the optimal cumulative dose (the total dose of rapamycin taken over the course of treatment) of rapamycin in the studied group of patients **(B)** resulting from mathematical modeling. Only variables related to rapamycin dosage were consistently and clearly associated with the model performance. Detected feature importance from autoML model **(A)** and partial-dependence plots **(B)** showed non-linear relationship between therapeutic concentration of rapamycin and total cumulative dose of the drug taken during given period of therapy. The ratio of seizure reduction within these dependencies, expressed as average prediction. We used the DALEX package (30) to find relationship between individual variables in ML model and predicted outcome. First, we used it to calculate feature importance. In plot 3 (“feature importance”), one can see average drop in classifier accuracy when variable was permuted (scrambled). Higher loss meant higher importance. Then, we calculated partial dependence plots for two most important variables (rapamycin dosage on previous visit and cumulative rapamycin dosage), plots on the right hand side showed the average prediction of ML model given different levels of those variables.

Figure 3 presents the outcome of the mathematical analyses. With the optimal model performance only the variables related to rapamycin dosage were consistently and clearly associated. DP analysis further indicated that their relationships with the outcome were non-linear—in both cases, small values of variables were associated with highest predicted improvement. One additional DF used Model #1 was compensated with enough improvement to obtain *p* < 0.001 when compared to the null model. Model #2 with six additional DFs was not significantly better than model #1 (*p* = 0.06). Same was true for models #3 vs. #2 (*p* = 0.46). Given that the study subjects had predominantly focal seizures with impaired awareness, there was no specific type of seizures most sensitive to rapamycin treatment. No influence of rapamycin on the semiology of epileptic seizures was observed. There was no significant relationship indicating the influence of age on the effectiveness of rapamycin treatment in the studied group of patients.

### Adverse Events

The safety profile of rapamycin obtained in the study group did not differ significantly from the information available in the literature (31) (Table 2). Respiratory tract infections were the most common adverse reactions in the study population (*N* = 15), followed by stomatitis (aphthous ulcers; *N* = 11). All of these adverse events were grade 1 or 2. No serious adverse events were observed in the study group. Upper respiratory tract infections were the cause of temporary drug withdrawal in 7 patients; however, with all of them rapamycin was restarted at the same dose. None of the patients required reduction of the rapamycin dosage due to adverse events. Importantly, the favorable profile of adverse events in the study group did not differ between the group of infants and the group of older children.

**Table 2:** Adverse events of any cause reported during the rapamycin treatment.

	**Rapamycin 0–6 months**		**Rapamycin 6–12 months**		**All**	
	**All grades**	**Grade 3–4**	**All grades**	**Grade 3–4**	**All grades**	**Grade 3–4**
Any adverse event	21	0	7	0	28	0
Stomatitis	8	0	3	0	11	0
Upper respiratory tract infection	12	0	3	0	15	
Diarrhea	0	0	0	0	0	0
Nasopharyngitis	0	0	0	0	0	0
Rash	0	0	0	0	0	0
Vomiting	0	0	0	0	0	0
Headache	0	0	0	0	0	0
Hypercholesterolemia, hypertrigliceridemia	0	0	1	0	1	0
Decreased appetite	1	0	0	0	1	0
Acne	0	0	0	0	0	0
Pharyngitis	0	0	0	0	0	0
Fatigue	0	0	0	0	0	0
Pneumonia	0	0	0	0	0	0

## Discussion

The results of the study presented here confirmed the satisfactory efficacy of rapamycin as an add-on therapy in TSC-related drug-resistant epilepsy. There was no possibility of a simple comparison of these results with the results of large, randomized studies with Everolimus. However, it seems appropriate to state that in both cases, the therapeutic effect, although satisfactory, was not associated with a spectacular and quick improvement. Theoretically, that was expected before the start of clinical trials with these drugs in view of their potential disease-modifying mechanism in the TSC population. Sixty five point 5 % of patients in the observation range achieved at least a 50% reduction in the frequency of epileptic seizures. The safety profile of the drug, also after prolonged use, proved to be satisfactory. Even after 12 months of use, side effects did not significantly impede the continuation of therapy. While the occurrence of low-grade side effects was common, which sometimes led to a temporary discontinuation of rapamycin treatment, the pattern in the population that we studied was relatively good drug tolerance. A satisfactory treatment tolerance and a favorable adverse event profile also applied to the youngest infants observed in this study. This observation may have significant clinical implications, given that the early initiation of an mTOR inhibitor may prove to be a therapy of the future in the treatment of TSC. It is clinically relevant to pinpoint the fact that the expected positive therapeutic effect was usually delayed. Therefore, and taking into account the potentially modifying effects of mTORi on many aspects of TSC, it seemed reasonable to maintain therapy for a longer time to achieve the clinical effect. The results presented in this paper did not in any way suggest that the use of a lower daily dose of rapamycin over a longer period was clinically justified. However, we would like to draw attention to the potentially beneficial effect of prolonged drug administration, even in the case of initial therapeutic failure. Another interesting finding in our study was an optimal concentration range in which the potentially best anti-epileptic effect of rapamycin was observed. This was important in minimizing the likelihood of side effects. The curvilinear relationship of clinical improvement depending on rapamycin concentration obtained in our group suggested an optimal concentration close to 4.5 mg/kg/day. On the other hand, the analysis of the cumulative dose of rapamycin and its efficacy showed linear association, suggesting the need for longer treatment. We found slightly better effects of treatment in the group with *TSC1* gene mutation which may be related to a generally better prognosis and a milder course of the disease in this group of patients. The results obtained in the study supported the efficacy of the combined use of vigabatrin and the mTOR inhibitor (in this case rapamycin) in the treatment of seizures associated with TSC. Whether it is related to the inhibitory effect of vigabatrin on the mTOR pathway, as suggested by some experimental studies (31), or on the contrary, on the additive effect on totally separate mechanisms of epileptic seizures, requires further research. It was important to note that in our study only the patients with a long history of drug resistant epilepsy, in whom multiple antiepileptic drugs had failed, were included in the study. There were no data on very early, let alone preventative, treatment with mTOR pathway inhibitors. It should be assumed that in such a case the effects would be more beneficial.

Our study is noted to have limitations. Although all subjects were tracked prospectively, the data have been collected in circumstances similar to everyday clinical practice. Thus, the study did not follow the methodological regime typical of RCTs. The number of seizures were reported by the patients' caregivers in the diaries used both before the introduction of rapamycin and during the study. All patients had a history of drug-resistant epilepsy and were followed in one epilepsy reference center. The caregivers were trained how to recognize seizures, but it cannot be excluded that some seizures might not have been counted. Another important issue to be addressed was improving the quality of life for patients. For the group of patients described here, we did not have a systematic database of quality-of-life questionnaires. Keeping this in mind, one might assume that, although on the whole, the reduction of more than 50% of the seizures occurred in over 60% of the subjects, the baseline frequency of the seizures as high as 8 seizures per week makes the reduction not entirely satisfactory. However, the hallmark of our patient group was that all of the participants had drug-resistant epilepsy after many past failures of pharmacological treatment. We believe that the obtained reduction in the frequency of seizures with a relatively favorable profile of adverse effects exerted by the studied drug, as well as the potentially beneficial effect of mTOR pathway inhibitors on various, also not related to the central nervous system, manifestations of tuberous sclerosis constitute sufficient justification for undertaking this type of therapeutic intervention. The benefit from the reduced number of seizures likely improved the quality of life of the patients and their families. A limitation that should also be noted was the relatively large age range in the study group.

Tuberous sclerosis is a model example of a genetically determined disease associated with symptomatic severe epilepsy starting in early infancy (1). The identification of the genes responsible for the development of TSC as well as their protein products and their key role in the activation of mTOR pathway in the pathogenesis of TSC led to disease modifying strategies (32). Two mTOR inhibitors, rapamycin and everolimus, after obtaining encouraging results in TSC animal models, were introduced in clinical trials and practice (33). Earliest reports concerned the treatment of renal AML and SEGA with rapamycin in small groups of patients diagnosed with TSC (21–23). In subsequent years, clinical studies were conducted primarily with the use of everolimus. Large randomized controlled trials have confirmed the effectiveness of this drug in the treatment of SEGA and AML associated with tuberous sclerosis (18, 19). Research on the use of mTOR inhibitors in the treatment of neurological symptoms of TSC, mainly epilepsy, followed a similar path. After the initial case reports regarding efficacy of both rapamycin and everolimus in the treatment of TSC-related epilepsy, subsequent randomized studies were only conducted with everolimus (20, 34). In 2013, the first prospective clinical trial to specifically evaluate everolimus efficacy for medically refractory epilepsy in patients with TSC was conducted (35). Seizure frequency was reduced by 50% or more in 12 of 20 patients, including several cases where dramatic improvement was observed in patients with a prior history of failed medications, vagus nerve stimulation, or epilepsy surgery. Improvement in multiple aspects of behavior and neurocognition were also reported. It is worth noting that response to treatment was highly variable, and three patients experienced an unexpected increase in seizures. Complicating the clinical picture were the phase III clinical trial results for SEGA (18, 36), in which seizure frequency and behavior measures as secondary endpoints failed to confirm the treatment benefit of everolimus. In 2017, everolimus was approved by FDA and EMA for the treatment of drug-resistant epilepsy associated with TSC and became the main mTOR inhibitor used in this indication. Despite the satisfactory antiepileptic efficacy of evrolimus, it is noteworthy that the results were not unequivocally or even spectacularly higher than those obtained with conventional antiepileptic drugs, which was expected given the potential disease-modifying effect of everolimus in TSC. Probably the key in this case was the early or even very early initiation of treatment and its consistent, long-term use: the latter suggestion also arising from the results obtained in this study. Everolimus is not widely available in many countries, including Poland. In our country, only rapamycin is reimbursed for epilepsy in TSC, even though the results of the clinical trial were not conclusive. At present, there are no uncontested comparative, face-to-face clinical or experimental data that confirm the superiority of one drug over another. Individual experimental works suggest the superiority of everolimus in neurological applications, but this requires further research (37). Curatolo et al. (36), in a *post-hoc* analysis of the phase 3 EXIST-3 trial with everolimus, reported that drug efficacy persisted, with sustained seizure reduction after 1 year of treatment across both pediatric subgroups [response rate 48.9% (95% CI 38.1–59.8)] for the younger subgroup [<6 years vs. 47.2% (39.3–55.2)] for the older subgroup (>6 years); thus, suggesting that everolimus appeared to be more effective in younger children. In our study, no such relationship was found.

In conclusion, in this open-label study, we observed the improvement in seizure control in drug-resistant epilepsy associated with TSC with rapamycin, especially in a longer follow-up. Considering also its favorable safety profile in TSC patients, rapamycin may be a potentially important therapeutic option, especially when everolimus is not available. However, further studies, especially controlled, randomized clinical trials are needed.

## Data Availability Statement

The raw data supporting the conclusions of this article will be made available by the authors, without undue reservation.

## Ethics Statement

The studies involving human participants were reviewed and approved by Komisja Bioetyczna przy Instytucie “Pomnik-Centrum Zdrowia Dziecka” w Warszawie. Written informed consent to participate in this study was provided by the participants' legal guardian/next of kin.

## Author Contributions

KSa, KK, and SJ contributed to conception and design of the study. KSa, DD-P, JB, DC, and AU organized the database. KSi performed the statistical analysis. KSa wrote the first draft of the manuscript. KK and KSi wrote sections of the manuscript. All authors contributed to manuscript revision, read, and approved the submitted version.

## Funding

The work was partially supported by the Polish ministerial fund for science (2013 to 2019) for the implementation of international co-financed project, Children's Memorial Health Institute grant no. 168/2018, and the grant EPIMARKER of the Polish National Center for Research and Development No. STRATEGMED3/306306/4/2016.

## Conflict of Interest

KSi was employed by company Transition Technologies S.A. The remaining authors declare that the research was conducted in the absence of any commercial or financial relationships that could be construed as a potential conflict of interest.

## Publisher's Note

All claims expressed in this article are solely those of the authors and do not necessarily represent those of their affiliated organizations, or those of the publisher, the editors and the reviewers. Any product that may be evaluated in this article, or claim that may be made by its manufacturer, is not guaranteed or endorsed by the publisher.
